# Vulval lichen sclerosus in UK general practice: a cross-sectional survey of patient experience

**DOI:** 10.1136/bmjopen-2025-103415

**Published:** 2025-09-05

**Authors:** Sophie Rees, Susanne Arnold, Helen Parsons, Sarah Hillman

**Affiliations:** 1 Bristol Trials Centre, University of Bristol, Bristol, UK; 2 University of Warwick Warwick Clinical Trials Unit, Coventry, England, UK; 3 University of Birmingham, Birmingham, UK

**Keywords:** DERMATOLOGY, Primary Health Care, GYNAECOLOGY, Quality of Life

## Abstract

**Abstract:**

**Objective:**

To explore experience and prevalence of vulval lichen sclerosus (VLS) diagnosis in general practice using an anonymous patient survey.

**Design:**

Quantitative descriptive cross-sectional survey informed by previous qualitative interviews and developed with patient representatives, sent to people recorded in general practice as having a VLS diagnosis.

**Setting:**

General practices (n=24) in the UK (West Midlands).

**Participants:**

n=177 respondents.

**Results:**

One in five respondents reported that they had been misdiagnosed, and about a third reported that it was a struggle to get treatment. Only one third said they received regular check-ups, recommended in clinical guidelines. One-fifth reported they were not being treated with topical corticosteroids, the main first-line treatment for VLS. Less than one in 10 were members of a support group, and around four in 10 felt they had to hide their condition and did not speak to anyone else about it. Survey respondents prioritised improving education and awareness among healthcare professionals (HCPs).

**Conclusion:**

General practitioners and other primary care HCPs have a key role in recognising, diagnosing and managing VLS. Improving education and awareness among HCPs was a key priority for this patient group. Patients should be made aware of the need for ongoing treatment and yearly check-ups to prevent or manage disease progression. VLS is a highly stigmatised condition, and appointments with HCPs may be the only opportunity for people to talk about their experience.

**STRENGTHS AND LIMITATIONS OF THIS STUDY**

- We gained insight into the variability of experience by recruiting a general practice population, many of whom were not members of support groups.
- A quantitative descriptive survey method did not allow probing of responses or exploration of unanticipated experiences.
- A lack of ethnic diversity in our respondents limits the representativeness of the results, although it potentially indicates an under-diagnosis of vulval lichen sclerosus in women of colour.

## Introduction

 Lichen sclerosus is a chronic inflammatory dermatological condition, typically affecting the anogenital skin. It can affect anyone at any age, but in this article, we are focusing on vulval lichen sclerosus (VLS) in adult women. Typical signs and symptoms include white or ashy patches in a ‘figure of eight’ shape around the vulva and perianal skin, pruritus, fissures and dyspareunia. VLS is a progressive condition, which can lead to scarring of the vulval skin and irreversible anatomical changes, and it is also associated with a higher risk of vulval cancer.^1^,^3^ First-line treatment is with ultrapotent topical corticosteroid (TCS) ointment, and guidelines recommend yearly check-ups to monitor treatment effectiveness and disease progression.^3^ VLS was previously thought to have a bimodal incidence in prepubertal girls and postmenopausal women, but recent research indicates that many women of reproductive age may have undiagnosed VLS.^1^

There is little evidence on the prevalence of VLS in general practice. Most research on incidence has been undertaken in secondary care. For example, examining a patient database at a general gynaecology practice in the US, 1.7% (n=28) had biopsy-proven VLS.^4^ A study published in 1996 found that 45% of NHS GPs saw more than one patient per month with recurrent vulval symptoms, with itch being the predominant symptom.^5^ In another study in Israel, researchers surveyed care home residents, finding that 3% had VLS.^6^ VLS is associated with urinary incontinence^7^ and therefore may be more common among older people and frail populations. A study of a registry in Finland estimated the incidence of VLS to be 1.6% by the age of 80.^8^ Any estimate of prevalence is likely to be an underestimate, as people may be too embarrassed to seek help, and when they do, healthcare professionals (HCPs) may not examine or recognise VLS.^3^

Vulval disease can have a profound impact on well-being. A survey found that one in five women with a vulval disorder had considered suicide or self-harm as a result of the condition.^9^ In a recent priority-setting exercise, understanding the impact of VLS on quality of life was a top 10 priority.^10^ Previous research on quality of life in VLS has tended to be quantitative in nature^11^ and often focused on sexual dysfunction,^12^,^17^ just one domain potentially affected by VLS. A systematic review found that 60% of women with VLS reported an impact on sexual dysfunction.^18^ Qualitative research about VLS is also limited.^19^ Ours was the first in-depth qualitative study of the lived experience of VLS.^20 21^ Little research has examined the experiences of VLS patients in general practice, and social research about vulval dermatoses has tended to recruit from secondary care clinics^22 23^ or online support groups.^20 24 25^

In this paper, we report the results of a patient survey conducted in general practice. We aimed to gain an understanding of the concerns and experiences of the general practice population, which may differ from those recruited from support groups. We also aimed to identify current unmet needs and challenges experienced by this patient population, to inform future research and intervention development. In the discussion, we consider the implications of these findings in the context of existing qualitative research.

## Methods

### Design

This was a cross-sectional survey of patients registered in general practices. After completing qualitative analysis of 20 interviews about the lived experiences of women with VLS, we identified key aspects of living with VLS, which participants had expressed and which we felt were present across much of our sample.^20 21^ We used this analysis to develop the statements for the survey Likert scales and used verbatim language. See online supplemental material for a copy of the survey, which was 21 pages in total, including cover page and consent form with 50 items and five sections.

### Patient and public involvement

We established a patient advisory group made up of four women with VLS for the entire mixed-methods study. They supported the development of the funding application and study design, and we held regular meetings with them throughout the study. We met with this group to explore their views on the draft survey, and to identify other key areas for inclusion, such as when respondents had been diagnosed, which HCPs diagnosed them, and whether they were part of a support group. We asked the patient advisory group to fill in the draft survey and addressed any difficulties they raised or any confusing wording.

### Sampling and recruitment

Working with the local research delivery network, practices were approached about the survey, ensuring that general practices in urban and deprived areas were invited. Additionally, a small number of practices approached us, and we also used our professional networks to invite others. Searches of practice databases identified patients who were coded as having a diagnosis of VLS and aged 18 or over. Convenience sampling was used, with all patients meeting these criteria eligible to take part. As it was exploratory and we were unsure of how many patients we would identify from the searches, we were aiming for at least 100 responses, although we did not plan any complex statistical analyses. To mitigate non-response bias, we assured potential respondents that their responses would be confidential, did not ask for any identifiable information, provided a free return envelope for postal questionnaires, and offered both online and paper options to complete the survey. We encouraged practices to send out the survey to as many of their patients with VLS as possible, although they were able to exclude anyone the screening HCP thought inappropriate to contact (eg, recently bereaved, terminal illness). Patients with a mobile number registered and aged under 75 were sent a text message from the practice containing a link to the patient information leaflet (PIL) and online survey. Those without a mobile number or aged 75 or over were sent postal packs containing an invitation letter, PIL, survey and a pre-paid return envelope. The PIL also included a link to the online version of the survey. Those who were sent text messages could contact the study team to request a postal pack. The online survey was built using Qualtrics. To protect confidentiality, no personal data was collected in the survey, and return envelopes were labelled ‘Confidential’ and addressed directly to the research team members. Qualtrics is a secure platform, and the surveys were downloaded and removed from the online platform at the end of the recruitment period, and any identifiable data entered as free text was removed.

### Data collection and analysis

The survey was completely anonymous. Participants completed the survey online via the link or QR code, or on paper returned by post. The consent form was embedded in the first page of the survey, and no identifiable details were collected. Data was entered into a SPSS (V.29) database by SA. Analysis was descriptive and conducted using SPSS by SR and HP. We shared the findings with the patient advisory group and discussed, which were of particular importance to them (eg, support group membership, speed to diagnosis, check-ups).

## Results

We first present the results of the practice searches (prevalence) and demographics of the participants. Following this is an analysis of the survey responses regarding diagnosis, treatment and follow-up, and the impact on everyday life.

### Search results

Twenty-four general practices took part in the survey. All were in the West Midlands and Warwickshire and of varying size (mean list size 11 930; range 3528–44 726). We included practices located in postcodes with a wide range of deprivation deciles. The mean deprivation decile was 6, with a range of 2–9. On average, practice populations were 95% white (range 88%–99%), and 76% of patients reported a positive experience of the practice (range 44%–92%) (online supplemental Table S1).^26^ In England and Wales, 82% of the population identifies as white, and in the West Midlands, this is lower, at 77%. This indicates that the practice populations were much less ethnically diverse than the national and regional average.

**Figure 1:**
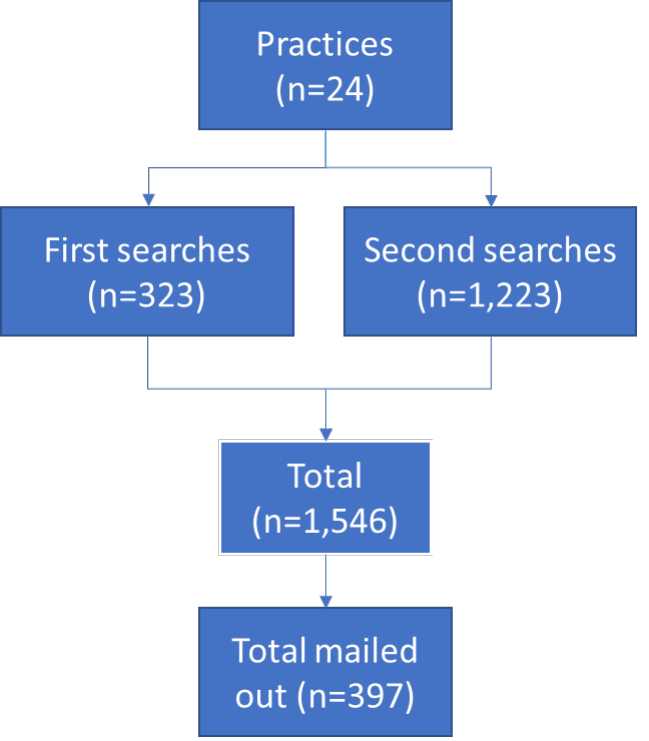
Searches and mailouts.

Practice searches identified 1546 patients coded with VLS (figure 1). Mailouts took place between July 2022 and February 2023, and the survey was open until 31 July 2023.

After searches and mailouts were completed at 12 practices, we discovered that a key term (*‘lichen sclerosus et atrophicus’*) had been omitted from the original search. We amended this for the next 12 practices, and we also returned to earlier practices to gain accurate search data (shown in figure 1 as ‘Second searches’). This resulted in a much greater number of patients identified (see figure 1); however, only one of these practices agreed to send a second mailout; therefore, we did not approach all eligible patients at eleven practices. Although the ‘et atrophicus’ suffix is outdated terminology now rarely used by specialists, it clearly remains a key code for general practice.

A mean of 67 patients per practice was identified (range 12–207), which corresponds to 0.58% (range 0.28%–1.08%) of the total practice list (including men and children) having a diagnosis of VLS.

### Participants

We received a total of 177 responses to the survey (45% response rate). We included partially completed survey responses in our analysis.

The mean age of respondents was 53 years (range 23–72) (table 1). Mean years since diagnosis was six (range <1 to 23), and 98% (n=173) of respondents identified as white.

**Table 1:** Respondent age group as reported in the survey

Age group	Frequency (n=176 total*)	Per cent of total
20–44	4	2
45–54	14	8
55–64	47	27
65–74	60	34
75–84	39	22
85 and over	12	7

* One respondent did not provide an answer.

Nearly all respondents identified as white cisgender heterosexual women (online supplemental Table S2).

**Figure 2:**
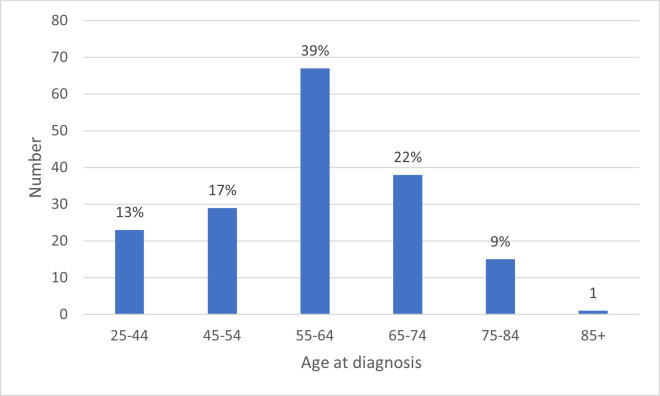
Age at diagnosis.

Figure 2 shows age *at diagnosis* reported in the survey, with the largest group being 55–64 (n=67, 39%).

### Diagnosis

‘Itching’ was the most commonly reported symptom (96%, n=160 at least sometimes), followed by burning (86%, n=142 at least sometimes) (online supplemental Table S3). Of 147 responders, 59% (n=87) stated they had experienced fusing or changes in architecture.

Ninety per cent (n=155) of respondents first sought help for their vulval symptoms from their GP, demonstrating that GPs and other practitioners in general practice play a key role in recognising and diagnosing VLS. Most commonly, VLS was diagnosed by GPs (n=78, 45%), followed by gynaecologists (n=55, 32%) and dermatologists (n=27, 16%).

Fifty-four per cent (n=92) reported being diagnosed within 6 months, and only 1 in five said they had been misdiagnosed (20%, n=34), which suggests that VLS is often recognised and diagnosed quickly in general practice. The most reported misdiagnosis was thrush (n=26). However, 33% (n=53) agreed with the statement *‘I feel that lack of healthcare professional knowledge about VLS delayed my diagnosis’*, and 25% (n=42) reported it took longer than 12 months to be diagnosed.

### Treatment and follow-up

Table 2 summarises the results related to treatment. Seventy-nine per cent (n=132) of respondents were currently using TCSs. Of these, 70% (n=92) were using TCSs reactively (in the event of a flare), and 30% (n=40) proactively (regularly even in the absence of symptoms). Most of these were using an ultrapotent steroid (n=87, 70%). Usually, participants reported that they based this regimen on HCP advice (70%, n=91). Most respondents (80%, n=104) felt ‘extremely confident’ or ‘slightly confident’ using their treatment. Only eight (6%) said they felt unconfident.

**Table 2:** Treatment reported by respondents

Characteristic	Category	N (%)
Current steroid treatment	Yes	132 (79)
	No	35 (21)
Steroid potency	Ultrapotent	87 (70)
	Potent	13 (11)
	Moderate	14 (11)
	Mild	7 (6)
	Not sure	3 (2)
Regimen	Proactive (even between flares)	40 (30)
	Reactive (only when symptoms flare)	92 (70)
How they chose this regimen	Advised by a healthcare professional	91 (69)
	Trial and error	5 (4)
	Other	35 (27)
Confidence in using the treatment	Extremely confident	73 (56)
	Slightly confident	31 (24)
	Neither confident nor unconfident	19 (14)
	Slightly unconfident	5 (4)
	Extremely unconfident	3 (2)

Most of the survey respondents (69%, n=112) reported they were given enough detail about how to use their treatment (online supplemental Table S4). However, one third (32%, n=52) agreed with the statement *‘It is a struggle for me to get the right treatment’*.

Only 33% (n=53) reported they have a regular check-up, despite this being recommended in expert guidelines.^3^ Of these, 48% (n=26) have check-ups with their GP, with the rest evenly split between dermatologists and gynaecologists. A quarter of survey respondents (25%, n=30) agreed with the statement ‘I have felt dismissed by HCPs when seeking help for my LS’ (online supplemental Table S4).

### Impact on everyday life

As described, we asked respondents to indicate their agreement with statements about the impact on quality of life, which were developed based on our qualitative study (online supplemental Table S5).^20^ In the survey, 55% (n=85) agreed that VLS is a ‘high maintenance’ condition, but 51% (n=69) disagreed that they have had to make changes to their everyday lives. Over three-quarters of respondents (77%, n=120) agreed that their VLS has become part of their everyday routine, and 62% (n=96) feel limited or restricted because of their VLS. About a fifth (21%, n=31) reported feeling lonely or isolated because of their VLS, and 43% (n=63) felt they needed to hide their VLS. Forty-three per cent (n=75) of survey respondents said they don’t talk to anyone about their VLS (online supplemental Table S6). Just 7% (n=11) of respondents said they were a member of a support group.

The impact on respondents’ sexual function and experiences was clear (see online supplemental Table S7). Sixty-six per cent (n=69) agreed they are not ready to give up the pleasure related to sex, and 72% (n=68) felt guilty about being unable to have sex. However, psychosexual counselling was voted the lowest priority from a list of research priorities by the survey respondents (online supplemental Table S8).

## Discussion

### Summary and comparison with existing literature

This study has surveyed VLS patients in general practice and reports prevalence data using searches of GP records. We found that VLS is not particularly rare in general practice, affecting a mean of 0.6% of all listed patients. This is likely an under-estimate as many people with VLS may not seek help due to embarrassment and, when they do, they may not be examined and VLS may not be recognised, and it may not be coded even if recognised. These challenges may be even more acute within disadvantaged groups such as those from minority ethnic communities. General practice has a key role in the diagnosis and management of VLS, as the first place most patients seek help.

In the search results, numbers were highest in the aged 60 and over categories. This reflects long-held assumptions that VLS is more common in postmenopausal women, although recently it has been suggested that many younger women may have undiagnosed VLS.^1^ Our study provides evidence of the prevalence of VLS as 0.6% of all patients in general practice, which suggests that around 1%–1.2% of women or those assigned female at birth are registered with VLS in general practice. Previous research found VLS to be present in 1 in 30 women living in a residential care home,^6^ and a nationwide registry study estimated prevalence by age 80 to be 1.6%.^8^ It is likely that the incidence of VLS rises with age since urinary incontinence is thought to be associated.^7^

Around 80% of respondents reported that they were currently using TCSs to treat their VLS, and around two-thirds of these reported using ultrapotent TCSs. Ultrapotent TCSs are the first-line recommended treatment for VLS,^3^ and our study indicates some patients in general practice could be being undertreated, which has implications for disease progression and risk of vulval cancer. Where qualitative research reports that women use trial and error and information from peers to inform their treatment approach,^20^ most of our survey respondents reported that their treatment routine was recommended by a clinician.^27^

The survey respondents prioritised improving HCP knowledge and understanding, which is also a theme in qualitative research.^20 24 25^ Recent research with HCPs highlights limited knowledge and understanding about vulval dermatoses,^27^ although in our survey results, patients reported being diagnosed relatively quickly. Resources have been produced to support HCP understanding.^28^,^30^ Guidelines on VLS recommend yearly follow-up to check disease progression and treatment use,^3^ but most participants in our survey were not receiving this. Qualitative research indicates that patients find regular check-ups reassuring.^20^ We believe that many patients managed in general practice may be unaware of the need to continue their treatment long-term or to regularly self-examine and receive yearly check-ups to prevent or manage disease progression. Patients should be reminded that they can request a yearly check-up with their HCP.

HCPs should ask about the impact on sexual function. Psychosexual counselling has been demonstrated to improve the quality of life with VLS.^31^ Many respondents were not ready to give up on the idea of sexual enjoyment and may be open to counselling. However, it should also be noted that psychosexual counselling was voted the lowest research priority by the survey respondents.

Qualitative reports illustrate the profound impact on quality of life, including on everyday activities such as toileting and exercise, as well as sexual function and a sense of loneliness and isolation.^20 21 24 25^ The survey responses showed that many respondents felt restricted or limited in some way, but only around a fifth reported feeling lonely or isolated because of their VLS. Qualitative research has highlighted loneliness as a key dimension of VLS and the subsequent impact of this,^21^ but the survey suggests this is variable. Additionally, only a small minority of the survey respondents reported they were a member of a support group, which indicates that a general practice survey was an effective way to reach a group of patients with different experiences than those who may engage with support groups and volunteer for qualitative research. However, over 40% reported they felt they needed to hide their VLS, and a similar proportion said they did not talk to others about their condition, which converges with qualitative reports about shame and silence around vulval disorders.^21 25^

Our study has highlighted that experiences of VLS are highly variable, and overall experiences of diagnosis may be more positive than initial qualitative research indicated, although education of HCPs was still a high priority for our respondents. It is troubling that most patients were not receiving yearly follow-up, and many may be being undertreated, and this should be addressed in future work. Importantly, VLS is not rare in general practice, with around 1%–1.2% of women affected. Educational interventions for general practitioners and other general practice HCPs should focus on clarifying the guidelines around treatment and check-ups, but also support an increased understanding of the psychosocial challenges of living with VLS and the support and resources available to patients.

### Strengths and limitations

In contrast to our previous qualitative research, which recruited from VLS support groups, mailing lists and social media, our survey respondents were overwhelmingly not members of support groups. Our findings have illuminated some of the divergences and similarities between these groups, highlighted in our discussion. This method enabled us to gather a wide variety of experiences, which is perhaps more representative of the variable experiences of VLS than qualitative research alone. However, qualitative research enables probing and in-depth exploration of experience. Although we worked with our patient representatives to design the survey, respondents may have interpreted questions differently.

As the survey was anonymous, we were unable to link responses to individual practices. As a result, we do not know if all practices were represented in the responses, or whether the majority came from patients in a small number of practices with GPs with a special interest in women’s health or vulval disease, which would call into question the representativeness of their experiences. People from minority ethnic groups were severely under-represented in our survey, despite recruiting from an ethnically diverse part of the UK. Future research could consider other methods, or using language in recruitment materials that specifically mentions that contributions from people of minority ethnic backgrounds are sought and valued. The limited and unreliable ethnicity data recorded in general practice make it challenging to focus recruitment on minority ethnic groups when doing this type of research, and we did not collect the ethnicity of patients in our search results. Nevertheless, future research should focus on inequalities between groups in terms of VLS diagnosis, treatment, quality of life and follow-up.

### Implications for research

Research is needed to understand the experiences of those from minority ethnic backgrounds, as well as LGBTQIA+, disabled, older and neurodiverse women. As research in vulval disorders grows, these groups need to be included. The survey response rate was high (45%). This highlights that this population is willing to participate in research, including when conducted through their GP practice. Future research on VLS in general practice should be sure to include ‘lichen sclerosus et atrophicus’ terminology in any practice searches.

### Implications for practice

VLS is not rare in general practice, likely affecting around 1% of women, and awareness and understanding among HCPs needs to be increased to improve patient experience. HCPs should consider diagnoses other than yeast infections and genitourinary symptoms of menopause,^28^ and VLS patients should be given sufficient information about vulval care and treatment and informed of the need for yearly follow-up. Information about self-examination and self-management is available,^30 32^ as well as guidelines and resources for HCPs.^3 28 29^ Healthcare appointments (both primary and secondary) may be the only opportunity for patients to express their experiences and talk about their condition, and HCPs should be mindful of this.

## Supplementary material

10.1136/bmjopen-2025-103415 online supplemental file 1

10.1136/bmjopen-2025-103415 online supplemental file 2

## Data Availability

Data are available upon reasonable request.
