# Supplementary material for: Vulval lichen sclerosus in UK general practice: a cross-sectional survey of patient experience
Source: BMJ Open. 2025 Sep 5;15(9):e103415. doi: 10.1136/bmjopen-2025-103415 (PMC12414219; doi:10.1136/bmjopen-2025-103415)
Supplement: online supplemental file 1 [file bmjopen-15-9-s001.docx]

# Supplementary information

## Practice information

Table S1 - Practice information from PHE National General Practice Profiles

| **Characteristic** | **Mean (range)** |
| --- | --- |
| Deprivation decile *(1=most deprived, 10=least deprived)* | 6 (2-9) |
| % White patients | 95 (88-99) |
| % patients reporting a positive experience of the practice | 76 (44-92) |

## Survey demographics

Table S2 - Demographics of survey respondents

| **Characteristic** | **Category** | **N (%)** |
| --- | --- | --- |
| Gender | Female | 176 (99) |
|  | Prefer not to say | 1 (1) |
| Trans status | The same gender as assigned at birth | 175 (99) |
|  | Prefer not to say | 2 (1) |
| Sexual orientation | Heterosexual | 173 (98) |
|  | Other | 2 (1) |
|  | *Missing* | *2 (1)* |
| Ethnicity | White | 175 (98) |
|  | Asian/Asian British | 1 (1) |
|  | Prefer not to say | 1 (1) |
| Partner/spouse | Yes | 137 (77) |
|  | No | 38 (22) |
|  | Prefer not to say | 2 (1) |
| Biopsy-diagnosed | No | 106 (61) |
|  | Yes | 52 (30) |
|  | Do not know | 16 (9) |
|  | *Missing* | *3* |

Figure S1 - Comparison of ages in search and survey (percentages)

## Clinical signs and symptoms

Table S3 - Symptoms reported in the survey. (Percentages are of valid responses for each item, except for rightmost column which gives the percentage of responses in the sample)

| **Symptom** | **Always** | **Often** | **Sometimes** | **Never** | **No. of responses**  **(% of sample)** |
| --- | --- | --- | --- | --- | --- |
| Itching | 52 (31%) | 70 (42%) | 38 (23%) | 7 (4%) | 167 (94%) |
| Burning | 20 (12%) | 55 (33%) | 67 (41%) | 23 (14%) | 165 (93%) |
| Thinning or frail skin | 39 (25%) | 48 (30%) | 45 (29%) | 26 (17%) | 158 (89%) |
| Pain | 21 (13%) | 36 (22%) | 70 (43%) | 35 (22%) | 162 (92%) |
| Cuts/tears/splits | 17 (11%) | 42 (27%) | 58 (37%) | 38 (25%) | 155 (88%) |
| Fusing/changes in architecture | 25 (17%) | 24 (16%) | 38 (26%) | 60 (41%) | 147 (83%) |

## Healthcare experiences

Table S4 - Experiences of healthcare for VLS

|  | **Completely**  **agree** | **Somewhat**  **agree** | **Neither agree nor disagree** | **Somewhat**  **disagree** | **Completely**  **disagree** | **No. of responses (% of sample)** |
| --- | --- | --- | --- | --- | --- | --- |
| **I feel healthcare professionals took me seriously when seeking help for my LS** | 84 (52%) | 36 (22%) | 17 (10%) | 12 (7%) | 14 (0%) | 163 (92%) |
| **I feel that lack of healthcare professional knowledge about LS delayed my diagnosis** | 23 (14%) | 30 (19%) | 27 (17%) | 15 (9%) | 66 (41%) | 161 (91%) |
| **I was given enough information about LS when I was diagnosed** | 53 (33%) | 42 (26%) | 20 (12%) | 32 (20%) | 16 (10%) | 163 (92%) |
| **I have felt dismissed by healthcare professionals when seeking help for my LS** | 18 (11%) | 22 (14%) | 18 (11%) | 26 (16%) | 77 (48%) | 161 (91%) |
| **I had to fight to get the right diagnosis** | 13 (8%) | 13 (8%) | 31 (19%) | 19 (12%) | 84 (53%) | 160 (90%) |
| **I was given enough detail about how to use my treatment** | 70 (43%) | 42 (26%) | 11 (7%) | 21 (13%) | 19 (12%) | 163 (92%) |
| **It is a struggle for me to get the right treatment** | 15 (9%) | 37 (23%) | 27 (17%) | 21 (13%) | 63 (39%) | 163 (92%) |

## Impact on everyday life

Table S5 - Impact on everyday life. Percentages are of valid responses for each item, except for rightmost column which gives the percentage of responses in the sample)

|  | **Completely**  **agree** | **Somewhat**  **agree** | **Neither agree nor disagree** | **Somewhat**  **disagree** | **Completely**  **disagree** | **No. of responses**  **(% of sample)** |
| --- | --- | --- | --- | --- | --- | --- |
| **I feel limited or restricted because of my LS** | 38 (25%) | 58 (37%) | 14 (9%) | 17 (11%) | 28 (18%) | 155 (88%) |
| **I am able to forget about my vulval LS for long periods of time** | 26 (17%) | 41 (26%) | 22 (14%) | 32 (20%) | 36 (23%) | 157 (89%) |
| **I feel that I have to hide the fact that I have vulval LS** | 38 (25%) | 25 (17%) | 26 (17%) | 14 (9%) | 48 (32%) | 151 (85%) |
| **My vulval LS has become**  **part of my daily routine and everyday life** | 77 (49%) | 43 (28%) | 13 (8%) | 8 (5%) | 15 (10%) | 156 (88%) |
| **I feel less like myself**  **because of my vulval LS** | 32 (21%) | 43 (28%) | 27 (18%) | 12 (8%) | 40 (26%) | 154 (87%) |
| **Vulval LS is a high**  **maintenance condition** | 37 (24%) | 48 (31%) | 24 (16%) | 23 (15%) | 22 (14%) | 154 (87%) |
| **I feel lonely or isolated**  **because of my vulval LS** | 9 (6%) | 22 (15%) | 28 (19%) | 23 (15%) | 69 (46%) | 151 (85%) |
| **I have had to make changes to my everyday life as a result of my vulval LS** | 30 (19%) | 31 (20%) | 25 (16%) | 23 (15%) | 46 (30%) | 155 (88%) |

## Talking to others

Table S6 - Who do you talk to you about LS? (select all that apply)

| **Response** | **Number (%)** |
| --- | --- |
| **I don’t talk to anyone** | 75 (47%) |
| **Partner/spouse** | 55 (34%) |
| **Family member(s)** | 16 (10%) |
| **Friend(s)** | 16 (10%) |
| **Other** | 10 (6%) |
| **Total responses** | **161 (91%)** |

## Impact on sex

Table S7 - Impact on sexual relationships. Percentages are of valid responses for each item, except for rightmost column which gives the percentage of responses in the sample) In the survey, many respondents chose ‘not applicable’ for many of the sex-related questions (20-47%). This could be because they felt uncomfortable answering them, or because they have no sexual relationship, perhaps due to VLS or other reasons. We have removed the ‘not applicable’ responses

|  | **Completely**  **agree** | **Somewhat**  **agree** | **Neither agree nor disagree** | **Somewhat**  **disagree** | **Completely**  **disagree** | **No. of responses (% of sample)** |
| --- | --- | --- | --- | --- | --- | --- |
| **I worry about how having sex will impact on my vulval LS** | 63 (55%) | 26 (23%) | 11 (10%) | 8 (7%) | 7 (6%) | 115 (65%) |
| **I find other ways to be intimate with my partner(s)** | 26 (25%) | 30 (29%) | 24 (23%) | 10 (10%) | 14 (14%) | 104 (59%) |
| **I spend a lot of time on preparation and/or after care before and/or**  **after sex** | 16 (19%) | 17 (20%) | 21 (25%) | 17 (20%) | 13 (16%) | 84 (47%) |
| **I feel guilty that I am unable to fulfil that part of our relationship** | 47 (43%) | 21 (19%) | 18 (17%) | 8 (7%) | 15 (14%) | 109 (62%) |
| **I am no longer scared of having sex** | 13 (12%) | 15 (14%) | 19 (18%) | 19 (18%) | 39 (37%) | 105 (60%) |
| **LS has taken away the spontaneity of sex** | 53 (47%) | 32 (28%) | 13 (12%) | 6 (5%) | 9 (8%) | 113 (64%) |
| **It is important to have an understanding partner** | 109 (85%) | 10 (8%) | 6 (5%) | 2 (2%) | 1 (1%) | 128 (72%) |
| **Sometimes you just want to get sex over and done with** | 39 (41%) | 18 (19%) | 21 (22%) | 8 (8%) | 10 (10%) | 96 (54%) |
| **I feel I am missing out compared to other people** | 42 (37%) | 31 (27%) | 19 (17%) | 5 (4%) | 17 (15%) | 114 (64%) |
| **I am not ready to give up that pleasure in my life** | 45 (43%) | 24 (23%) | 12 (11%) | 10 (10%) | 14 (13%) | 105 (59%) |

## Research priorities

Table S8 Table of areas of research in order of importance given by respondents (higher numbers -> lower priority)

| **Area** | **Sum of ranks** | **Mean of ranks** |
| --- | --- | --- |
| **Education and awareness for healthcare professionals** | 464 | 2.62 |
| **Education and awareness for patients** | 491 | 2.77 |
| **The causes of vulval LS** | 537 | 3.03 |
| **Improvements in diagnosis** | 539 | 3.05 |
| **Preventing fusing and loss of architecture** | 642 | 3.63 |
| **Steroid use or alternatives to steroids** | 685 | 3.87 |
| **Issues with sex** | 777 | 4.39 |
| **Counselling or psychological help** | 838 | 4.73 |
