# Supplementary material for: Vulval lichen sclerosus in UK general practice: a cross-sectional survey of patient experience
Source: BMJ Open. 2025 Sep 5;15(9):e103415. doi: 10.1136/bmjopen-2025-103415 (PMC12414219; doi:10.1136/bmjopen-2025-103415)
Supplement: online supplemental file 2 [file bmjopen-15-9-s002.pdf]

Living with Vulval Lichen Sclerosus (LS) Survey

Researchers:

Dr Sophie Rees

Dr Susanne Arnold

Telephone: 024 7652 3355

livingwithLS@warwick.ac.uk

INTRODUCTION

Living with Vulval Lichen Sclerosus (LS) Survey.

Thank you for your interest in this survey study.

Please read the detailed Participant Information Leaflet **V3.0 dated 15/12/2021** enclosed with these documents or using this link XXX before deciding to take part.

Once you have read the information leaflet please proceed to the consent questions which you must answer before completing the survey.

If you would like further information about this research project, please contact

Dr Sophie Rees or Dr Susanne Arnold on 024 7652 3355 or email us at:

livingwithLS@warwick.ac.uk

PLEASE NOTE IF ANY OF THE QUESTIONS IN THIS SURVEY CAUSE YOU ANY DISTRESS OR CONCERN PLEASE CONTACT YOUR GP TO DISCUSS YOUR WORRIES.

SECTION 1: CONSENT

You must answer **YES** to questions **1-5** for your data to be included in the survey results.

Q1. I confirm that I have read and understand the information leaflet (Version 3.0 dated 15/12/2021) for this study. I have had the opportunity to consider the information. I have had the opportunity to ask questions and have them answered to my satisfaction.

No ☐

Yes ☐

Q2. I understand that my participation is voluntary. I understand that because the survey is totally anonymous, I am unable to withdraw my answers once I have submitted them.

No ☐

Yes ☐

Q3. I understand that data collected during the study will only be looked at by the research team at the University of Warwick where it is relevant to my taking part in this study or by authorised representatives of the University of Warwick for auditing or monitoring purposes. I give permission for these individuals to have access to my anonymous data.

No ☐

Yes ☐

Q4. I give permission for anonymous quotes from my survey to be used in the reporting of the study findings.

No ☐

Yes ☐

Q5. I agree to take part in this survey study.

No ☐

Yes ☐

Q6. I am happy for my anonymous data to be used in future research **(optional).**

No ☐

Yes ☐

SECTION 2: ABOUT YOU

Q1. How old are you?

- 18 - 24 ☐
- 25 - 34 ☐
- 35 - 44 ☐
- 45 - 54 ☐
- 55 - 64 ☐
- 65 - 74 ☐
- 75 - 84 ☐
- 85 or over ☐
- Prefer not to say ☐

Q2. With which gender do you identify?

- Female ☐
- Male ☐
- Non-binary/third gender ☐
- Prefer not to say ☐

Q3. I would describe my gender as:

- The same gender originally assigned at birth ☐
- Different to the gender originally assigned at birth ☐
- Prefer not to say ☐

Q4. I would describe my sexual orientation as:

- Heterosexual ☐
- Bisexual ☐
- Gay woman/lesbian ☐
- Gay man ☐
- Other ☐
- Prefer not to say ☐

Q5. What is your ethnic group?

- White ☐
- Mixed/Multiple Ethnicity ☐
- Asian/Asian British ☐
- Black/African/Caribbean/Black British ☐
- Other Ethnic Group ☐
- Prefer not to say ☐

Q6. Do you have a partner/spouse?

- No *(move to Q8.)* ☐
- Yes ☐
- Prefer not to say *(move to Q8.)* ☐

Q7. Do you live with your partner/spouse?

- No ☐
- Yes ☐
- Prefer not to say ☐

SECTION 3: DIAGNOSIS, SYMPTOMS AND TREATMENT FOR VULVAL LS

Q8. When you began experiencing your symptoms of vulval LS, where did you seek help? *(select all that apply).*

- GP ☐
- Sexual Health Clinic ☐
- Private Dermatologist or Gynaecologist ☐
- Did not experience symptoms ☐
- Other *(please specify)* ☐

Q9. How long did it take to get a diagnosis of vulval LS? *(if not sure, please give your best guess).*

- Less than 6 months ☐
- 6 - 12 months ☐
- More than 1 year ☐

Q10. How old were you when you were diagnosed with vulval LS? *(if not sure, please give your best guess).*

- 18 - 24 ☐
- 25 - 34 ☐
- 35 - 44 ☐
- 45 - 54 ☐
- 55 - 64 ☐
- 65 - 74 ☐
- 75 - 84 ☐
- 85 or over ☐
- Prefer not to say ☐

Q11. Who diagnosed your vulval LS?

- GP ☐
- Dermatologist ☐
- Gynaecologist ☐
- Sexual Health Clinic ☐
- Nurse ☐
- Not sure ☐
- Other *(please state who)* ☐

Q12. Was a biopsy taken to confirm your vulval LS?

- No ☐
- Yes ☐
- Do not know ☐

Q13. Was your vulval LS ever misdiagnosed as something else?

- No *(go to Q14)* ☐
- Yes ☐
- Not sure ☐

Q13a. Please choose what your vulval LS was misdiagnosed as? *(select all that apply).*

- Thrush ☐
- Sexually Transmitted Infection (STI) ☐
- Menopause ☐
- Other *(please specify)* ☐

Q14. These are things that people with vulval LS have told us are important.

Please choose how much you agree or disagree with each statement:

	Completely disagree	Somewhat disagree	Neither agree nor disagree	Somewhat agree	Completely agree
I feel healthcare professionals took me seriously when seeking help for my LS					
I feel that lack of healthcare professional knowledge about LS delayed my diagnosis					
I was given enough information about LS when I was diagnosed					
I have felt dismissed by healthcare professionals when seeking help for my LS					
I had to fight to get the right diagnosis					
I was given enough detail about how to use my treatment					
It is a struggle for me to get the right treatment					

Q15. Which, if any, of these symptoms of vulval LS have you ever experienced and how often?

	Never	Sometimes	Often	Always
Itching				
Pain				
Burning				
Cuts/tears/splits				
Thinning or frail skin				
Fusing/changes in architecture				
Other *(please specify)*				

Q16. Are you currently using steroid treatment for your vulval LS?

No *(go to Q17)* ☐

Yes ☐

Q16a. Which steroid are you using? *(select all that apply)*

Clobetasol Propionate (Dermovate) ☐

Clobetasol Butyrate (Eumovate) ☐

Mometasone Furoate (Elocon) ☐

Hydrocortisone ☐

Not sure ☐

Other *(please specify)* ☐

Q16b. When do you use your steroid treatment?

I only use it when I have symptoms/a flare *(go to Q16d.)* ☐

I use it even when I do not have symptoms ☐

Q16c. How often do you use your steroid treatment?

- Every day ☐
- Three times per week ☐
- Twice per week ☐
- Once per week ☐
- Other *(please specify)* ☐

Q16d. How did you decide on this routine for using your steroid treatment?

- Advised to do it this way by a healthcare professional ☐
- I talked to other people with vulval LS ☐
- Trial and error ☐
- Other *(please specify)* ☐

Q16e. How confident are you in applying your steroid treatment?

- Extremely confident ☐
- Slightly confident ☐
- Neither confident nor unconfident ☐
- Slightly unconfident ☐
- Extremely unconfident ☐

Q17. Have you ever been given general information by a healthcare professional about vulval skin care? *(e.g. moisturisers, emollients, lubricants etc.)*

- No ☐
- Yes ☐
- Not sure ☐

Q18. Have you ever been referred to a Dermatologist or Gynaecologist for your vulval LS by your GP?

- No *(go to Q19)* ☐
- Yes ☐
- Not sure ☐

Q18a. Were you seen by a Dermatologist or Gynaecologist in a specialist vulval clinic?

- No ☐
- Yes ☐
- Not sure ☐

Q19. Do you have a regular check up with a healthcare professional for your vulval LS?

- No *(go to Q20)* ☐
- Yes ☐

Q19a. Who is this check up with?

- GP ☐
- Dermatologist ☐
- Gynaecologist ☐
- Other *(please specify)* ☐

Q20. Have you ever paid to see a healthcare professional privately about your vulval LS?

- No *(go to Q21)* ☐
- Yes ☐

Q20a. What was your motivation for seeking private healthcare? *(select all that apply)*

- To see an LS specialist ☐
- Avoid NHS waiting lists ☐
- For peace of mind ☐
- I have a private healthcare plan ☐
- Other (please specify) ☐

SECTION 4: QUALITY OF LIFE

Q21. These are things that people with vulval LS have told us are important about quality of life. Please choose how much you agree or disagree with each statement:

	Completely disagree	Somewhat disagree	Neither agree nor disagree	Somewhat agree	Completely agree	Not applicable
I feel or have felt limited or re-stricted because of my vulval LS						
I feel in control of my vulval LS						
I feel like less of a woman because of my vulval LS						
I am able to forget about my vulval LS for long periods of time						
I feel that I have to hide the fact that I have vulval LS						
My vulval LS has become part of my daily routine and everyday life						
I feel less like myself because of my vulval LS						
Vulval LS is a high maintenance condition						
I feel lonely or isolated because of my vulval LS						
I have had to make changes to my everyday life as a result of my vulval LS						

Q22. These are things that people with vulval LS have told us are important about sexual relationships. Please choose how much you agree or disagree with each statement

	Completely disagree	Somewhat disagree	Neither agree nor disagree	Somewhat agree	Completely agree	Not applicable
I worry about how having sex will impact on my vulval LS						
I find other ways to be intimate with my partner(s)						
I spend a lot of time on preparation and/or after care before and/or after sex						
I feel guilty that I am unable to fulfil that part of our relationship						
I am no longer scared of having sex						
LS has taken away the spontaneity of sex						
It is important to have an under-standing partner						
Sometimes you just want to get sex over and done with						
I feel I am missing out compared to other people						
I am not ready to give up that pleas-ure in my life						

Q23. Who do you talk to about your vulval LS? *(select all that apply)*

- I don't talk to anyone ☐
- Partner/spouse ☐
- Family member ☐
- Friend(s) ☐
- Other *(please specify)* ☐

Q24. Are you a member of a vulval LS support group (online or other)?

- No *(go to Q25)* ☐
- Yes ☐

Q24a. What do you get out of being part of a support group? *(select all that apply)*

- To research/find out more about the condition ☐
- To share my experiences ☐
- To pick up hints and tips from others about managing the condition ☐
- To find some reassurance ☐
- To talk to people in the same position as me ☐
- To help others ☐
- Other *(please specify)* ☐

SECTION 4a: The following questions are taken from a survey about quality of life with a vulval condition. When answering them, please think about your health over the past MONTH.

Q25. Over the past month how itchy and/or painful and/or stinging and/or burning has your vulval skin felt?

- Very much ☐
- A lot ☐
- A little ☐
- Not at all ☐

Q26. Over the past month, how often have you experienced any of the following: pain when urinating, heat intolerance, vaginal discharge, wetness?

- Very much ☐
- A lot ☐
- A little ☐
- Not at all ☐

Q27. Over the last month how embarrassed or self-conscious have you been because of your vulval skin symptoms?

Very much ☐
A lot ☐
A little ☐
Not at all ☐

Q28. Over the past month how much has your vulval skin impacted your body image or sense of self? *(For instance sense of femininity, feeling isolated, feeling different).*

Very much ☐
A lot ☐
A little ☐
Not at all ☐

Q29. Over the last month how distressed or anxious have you felt because of your vulval skin problem?

Very much ☐
A lot ☐
A little ☐
Not at all ☐

Q30. Over the last month how much has your vulval skin problem influenced your choice of clothing? *(For instance underwear, jeans, gym clothes).*

Very much ☐
A lot ☐
A little ☐
Not at all ☐

Q31. Over the last month how much has your vulval skin problem disturbed your sleep?

Very much ☐
A lot ☐
A little ☐
Not at all ☐

Q32. Over the last month how much has your vulval skin problem made it difficult for you to go shopping, look after yourself or your family, home and garden?

- Very much ☐
- A lot ☐
- A little ☐
- Not at all ☐

Q33. Over the last month how much has your vulval skin problem made it difficult for you to attend social or leisure engagements? *(For instance going out for dinner or bars, dating, sport, exercise class, gym).*

- Very much ☐
- A lot ☐
- A little ☐
- Not at all ☐

Q34. Over the last month how much has your vulval skin problem interfered with your ability to concentrate on work or study?

- Very much ☐
- A lot ☐
- A little ☐
- Not at all ☐

Q35. Over the last month how much has your vulval skin problem created problems with a partner or precluded you from pursuing a romantic relationship? *(For instance maintaining a relationship or finding a partner).*

- Very much ☐
- A lot ☐
- A little ☐
- Not at all/Not applicable ☐

Q36. Over the last month how much has your vulval skin problem interfered with your sex life? *(For instance decreased libido, decreased frequency of sex, pain with sex and/or enjoyment of sex).*

- Very much ☐
- A lot ☐
- A little ☐
- Not at all/Not applicable ☐

Q37. Over the last month how often have you felt distressed or worried about sex because of your vulval skin?

- Very much ☐
- A lot ☐
- A little ☐
- Not at all/Not applicable ☐

Q38. How often in the last month have you been worried about long-term health implications of your vulval skin condition? *(For instance concern about developing cancer or difficulties with fertility).*

- Very much ☐
- A lot ☐
- A little ☐
- Not at all ☐

Q39. Over the past month, how much of a problem has the treatment of your vulval symptoms been? *(For instance messy, time consuming, expensive, inconvenient).*

- Very much ☐
- A lot ☐
- A little ☐
- Not at all/Not applicable ☐

SECTION 4b: The following questions are taken from a survey about quality of life. Under each heading, please tick the **ONE** box that best describes your health **TODAY.**

Q40. MOBILITY

- I have no problems in walking about ☐
- I have slight problems in walking about ☐
- I have moderate problems in walking about ☐
- I have severe problems in walking about ☐
- I am unable to walk about ☐

Q41. SELF-CARE

- I have no problems washing or dressing myself ☐
- I have slight problems washing or dressing myself ☐
- I have moderate problems washing or dressing myself ☐
- I have severe problems washing or dressing myself ☐
- I am unable to wash or dress myself ☐

Q42. USUAL ACTIVITIES *(e.g. work, study, housework, family or leisure activities)*

- I have no problems doing my usual activities ☐
- I have slight problems doing my usual activities ☐
- I have moderate problems doing my usual activities ☐
- I have severe problems doing my usual activities ☐
- I am unable to do my usual activities ☐

Q43. PAIN/DISCOMFORT

- I have no pain or discomfort ☐
- I have slight pain or discomfort ☐
- I have moderate pain or discomfort ☐
- I have severe pain or discomfort ☐
- I have extreme pain or discomfort ☐

Q44. ANXIETY/DEPRESSION

- I am not anxious or depressed ☐
- I am slightly anxious or depressed ☐
- I am moderately anxious or depressed ☐
- I am severely anxious or depressed ☐
- I am extremely anxious or depressed ☐

SECTION 4b:

Q45.

- We would like to know how good or bad your health is **TODAY.**
- This scale is numbered from 0 to 100.
- 100 means the best health you can imagine.
  0 means the worst health you can imagine.
- Please mark an X on the scale to indicate how your health is TODAY.
- Now, write the number you marked on the scale in the box below.

YOUR HEALTH TODAY = ☐

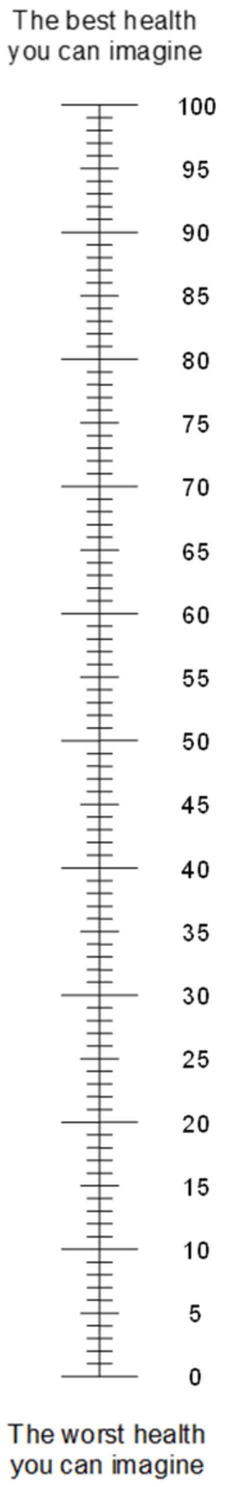

SECTION 5: FUTURE RESEARCH

Q46. Would you consider taking part in a clinical trial of a medical treatment for vulval LS?

- No ☐
- Maybe ☐
- Yes ☐
- Don't know ☐

Q46a. Please tell us more about your answer to Q46.

Q47. Would you be concerned about having photographs taken of your vulva for research purposes?

- No ☐
- Maybe ☐
- Yes ☐
- Don't know ☐

Q48. Would you consider taking part in a clinical trial about helping with self-managing vulval LS ?

- No ☐
- Maybe ☐
- Yes ☐
- Don't know ☐

Q48a. Please tell us more about your answer to Q48.

Q49. Please place these possible areas of research in order of importance *(write 1 next to most important, 2 next to second most important etc.)*

Counselling or psychological help	________
Education and awareness for patients	________
Education and awareness for healthcare professionals	________
Preventing fusing and loss of architecture	________
Improvements in diagnosis	________
Issues with sex	________
Steroid use or alternatives to steroids	________
The causes of vulval LS	________

Q50. Please confirm how you were approached to take part in this study.

- Text message ☐
- Invitation letter ☐

Thank you for completing this survey.

We really appreciate your time.

The results of this study will be available in the coming months on our website:

www.warwick.ac.uk/fac/sci/med/research/ctu/trials/lwvls/

IF ANY OF THE QUESTIONS IN THIS SURVEY HAVE CAUSED YOU ANY DISTRESS OR CONCERN PLEASE CONTACT YOUR GP TO DISCUSS YOUR WORRIES.
