# Supplementary material for: Vulval lichen sclerosus in UK general practice: a cross-sectional survey of patient experience
Source: BMJ Open. 2025 Sep 5;15(9):e103415. doi: 10.1136/bmjopen-2025-103415 (PMC12414219; doi:10.1136/bmjopen-2025-103415)
Supplement: Supplementary file 3 [file bmjopen-2025-103415.reviewer_comments.pdf]

PEER REVIEW HISTORY

BMJ Open publishes all reviews undertaken for accepted manuscripts. Reviewers are asked to complete a checklist review form and are provided with free text boxes to elaborate on their assessment. These free text comments are reproduced below.

ARTICLE DETAILS

Title (Provisional)

Vulval lichen sclerosus in UK general practice: a cross-sectional survey of patient experience

Authors

Rees, Sophie; Arnold, Susanne; Parsons, Helen; hillman, sarah

---

VERSION 1 - REVIEW

**Reviewer**	**1**
**Name**	**Kraus, Christina N**
**Affiliation**	**Department of Dermatology, University of California, Irvine, CA.**
**Date**	**21-May-2025**
**COI**	**None**

This is a nice study of VLS in primary care. It is thorough and well-written. A few suggestions: I would remove the reference to this being the "first" study in primary care as I do not think this adds to the paper. The study stands on its own merit, regardless of whether it is the first of its kind. As the definition of "primary care" differs by country and practice pattern, I would consider specifying what primary care practices were included here. Some countries consider gynecology to be "primary care". So if this was just limited to family medicine or internal medicine, I would specify. I would also include whether all primary care practitioners included were physicians or if some were PAs or NPs or other mid-level providers.

**Reviewer**	**2**
**Name**	**Paganelli, Alessia**
**Affiliation**	**IDI-IRCCS Dermopathic Institute of the Immaculate Conception**
**Date**	**25-May-2025**

**COI**	**None**

This manuscript tackles an important and often under-discussed issue—the experience of patients diagnosed with vulval lichen sclerosus (VLS) in primary care. The use of a patient-informed survey and collaboration with patient representatives is commendable and adds value to the study's relevance. However, there are a number of structural and clarity-related issues that, if addressed, could significantly enhance the overall quality and impact of the paper.

Introduction and Framing of the Study

At present, the introduction seems to focus quite early on the specific population surveyed, which may risk narrowing the reader's understanding before establishing a broader clinical or public health context. It would be helpful to begin with a more general overview of VLS—its prevalence, clinical significance, and current challenges in diagnosis and management—before narrowing the focus to this specific survey and population. Doing so would not only situate the study more effectively within the literature but also make the rationale clearer to a broader audience.

Clarifying the Study Aims

Currently, the study aims appear to be embedded within the methods section, which might confuse readers. These should instead be clearly presented at the end of the introduction. Separating the 'why' (aims) from the 'how' (methods) is important to preserve narrative clarity and better guide the reader through the logic of the paper.

Methods Section: Streamlining and Structure

The methods section is overly segmented into many sub-paragraphs. This makes it feel somewhat disjointed and harder to follow. A more cohesive structure—with perhaps one or two main methodological subsections—would improve readability. Moreover, some of the content, such as the identification of which author completed particular tasks, is not appropriate for the methods section. Such authorship contributions should be reserved for a dedicated statement at the end of the paper, rather than being integrated into methodological explanations.

Patient Involvement

Lines 109–110 mention that patients were involved in the research, but this needs greater specificity. How were they involved? Were patient advocacy groups or associations consulted? Did involvement take place in the survey design, piloting, or analysis stages? Providing a brief explanation here would help clarify the participatory nature of the study and strengthen its credibility.

Clarifying the Contribution and Implications

While the results clearly highlight key gaps in diagnosis, treatment, and follow-up care for VLS patients, the manuscript could more clearly articulate what new understanding this study brings. How does this survey differ from previous work in the field? More importantly, the conclusion mentions that improving education and awareness among primary care providers is a patient priority, but it remains somewhat unclear how the findings might concretely contribute to this goal. Strengthening the discussion around how these results could be translated into educational interventions or policy recommendations for general practitioners would make the paper's impact more tangible. Also, the discussion section should be enriched with comparisons with available literature

Last but not least Figure 1 should have a more catchy oulook to help the reader to follow the study workflow.

VERSION 1 - AUTHOR RESPONSE

Reviewer	Comment	Response
1	This is a nice study of VLS in primary care. It is thorough and well-written.	Many thanks to the reviewer for this comment
	I would remove the reference to this being the "first" study in primary care as I do not think this adds to the paper. The study stands on its own merit, regardless of whether it is the first of its kind.	We have changed the first line of the discussion to address this
	As the definition of "primary care" differs by country and practice pattern, I would consider specifying what primary care practices were included here. Some countries consider gynecology to be "primary care". So if this was just limited to family medicine or internal medicine, I would specify. I would also include whether all primary care practitioners included were physicians or if some were PAs or NPs or other mid-level providers.	We have edited the abstract, introduction and methods sections to make this clearer
2	This manuscript tackles an important and often under-discussed issue—the experience of patients diagnosed with vulval lichen sclerosus (VLS) in primary care. The use of a patient-informed survey and collaboration with patient representatives is commendable and adds value to the study's relevance.	We thank the reviewer for this comment.
	At present, the introduction seems to focus quite early on the specific	While we appreciate this comment, we have not revised the introduction

	population surveyed, which may risk narrowing the reader's understanding before establishing a broader clinical or public health context. It would be helpful to begin with a more general overview of VLS—its prevalence, clinical significance, and current challenges in diagnosis and management—before narrowing the focus to this specific survey and population. Doing so would not only situate the study more effectively within the literature but also make the rationale clearer to a broader audience.	because it currently presents the topic concisely and it does cover incidence, its impact on quality of life, and challenges around diagnosis in primary care. We think the introduction as it stands presents the rationale, and a longer general overview of VLS would not strengthen it.
	Currently, the study aims appear to be embedded within the methods section, which might confuse readers. These should instead be clearly presented at the end of the introduction. Separating the 'why' (aims) from the 'how' (methods) is important to preserve narrative clarity and better guide the reader through the logic of the paper.	We have added sentences describing the aims at the end of the introduction
	The methods section is overly segmented into many sub-paragraphs. This makes it feel somewhat disjointed and harder to follow. A more cohesive structure—with perhaps one or two main methodological subsections—would improve readability.	We have merged the data collection and analysis subsections of the methods
	Moreover, some of the content, such as the identification of which author completed particular tasks, is not appropriate for the methods section. Such authorship contributions should be reserved for a dedicated statement at the end of the paper, rather than being integrated into methodological explanations.	We wish to retain this information in the methods section, since transparency is important for reporting methods.
	Lines 109–110 mention that patients were involved in the research, but this needs greater specificity. How were they involved? Were patient advocacy groups or associations consulted? Did involvement take place in the survey design, piloting, or analysis stages? Providing a brief explanation here would help clarify the participatory nature of the study and strengthen its	We have expanded this to explain the role the patient advisory group played

	credibility.	
	While the results clearly highlight key gaps in diagnosis, treatment, and follow-up care for VLS patients, the manuscript could more clearly articulate what new understanding this study brings. How does this survey differ from previous work in the field?	We have added a short paragraph at the end of the discussion reiterating the new knowledge generated through our study
	More importantly, the conclusion mentions that improving education and awareness among primary care providers is a patient priority, but it remains somewhat unclear how the findings might concretely contribute to this goal. Strengthening the discussion around how these results could be translated into educational interventions or policy recommendations for general practitioners would make the paper's impact more tangible.	Although we agree this is important, we did not set out to generate educational interventions through this survey study. We have added a sentence at the end of the discussion to highlight the direction educational interventions should take
	Also, the discussion section should be enriched with comparisons with available literature	The discussion does contain many comparisons with existing literature and we are unsure where exactly we should increase this within the discussion
	Last but not least Figure 1 should have a more catchy oulook to help the reader to follow the study workflow.	We have edited the Figure to expand the labels and change the colours
